# Dental Histology

**Published:** 1885-03

**Authors:** 


### DENTAL HISTOLOGY.

The *Journal of the British Dental Association* for January, contains some severe strictures upon a paper read before the American Dental Association, at its last meeting, by Dr. J. L. Williams, of New Haven. We are of the opinion that had the editor known all the circumstances under which the paper was read, and the end in view in its production, he would not have written the article, for the *Journal* has never, to our knowledge, been accused of wanton unfairness.

A learned professor in one of our schools, a famous writer, and an acknowledged authority on most professional subjects, had read a paper before another body, in which he advanced some theories essentially identical with those taught by Goodsir, and the object of Dr. Williams was to combat these views, which, coming from such high authority, he deemed pregnant with evil, and misleading to students in histology. To this end he again went over the ground, reproducing old arguments and advancing new ones to prove that—

(*i*) The teeth are not developed as papillæ in a groove.

(*ii*) That there is an enamel organ with a definite function.

(*iii*) That the formation of enamel is from the line of its union with the dentine outward.

But he did not assert these as original discoveries of his own, although he exhibited some of his own microscopical sections in demonstration.

Concerning the fourth point that the editor of the *Journal* claims that Dr. Williams "actually pretends to foist on a credulous world as his own discovery," viz.:

(*iv*) That the odontoblasts are of the nature of ganglionic elements, sending processes outward into the dentine, backwards into the pulp, and probably laterally to each other—

The editor, in a preceding article in the same number, speaks of "the revolution effected by the observations of Heitzmann and Bodecker." Surely, he must be aware that Heitzmann and Bodecker have represented these same odontoblast cells as round or oval plastids, *without* prolongations. Klein and Smith, whom the editor will doubtless admit as competent English authority, have also recently represented the odontoblasts as oval or oblong cells, *without branches,* and the dental fibrillæ as springing from "special branched cells," which lie deeper in the pulp. So this long settled question does not seem to be settled at all, even according to the authorities quoted by the editor.

We are sorry to see such a matter dealt with in so flippant and undignified a manner. The levity of the article appears to us quite misplaced, and the writer has evidently failed to either understand Dr. Williams or the object that he had in view.

